# The prognostic significance of tumor-immune microenvironment in ascites of patients with high-grade serous carcinoma

**DOI:** 10.2478/raon-2023-0046

**Published:** 2023-11-30

**Authors:** Simona Miceska, Erik Skof, Simon Bucek, Cvetka Grasic Kuhar, Gorana Gasljevic, Spela Smrkolj, Veronika Kloboves Prevodnik

**Affiliations:** Department of Cytopathology, Institute of Oncology Ljubljana, Ljubljana, Slovenia; Faculty of Medicine, University of Ljubljana, Ljubljana, Slovenia; Department of Medical Oncology, Institute of Oncology Ljubljana, Ljubljana, Slovenia; Department of Pathology, Institute of Oncology Ljubljana, Ljubljana, Slovenia; Division of Gynaecology and Obstetrics, University Medical Centre, Ljubljana, Slovenia; Faculty of Medicine, University of Maribor, Maribor, Slovenia

**Keywords:** ascites, immune cells, high-grade serous carcinoma, PD-1, PD-L1, prognostic markers

## Abstract

**Background:**

High-grade serous carcinoma (HGSC) is often associated with ascites at presentation. Our objective was to quantify immune cells (ICs) in ascites prior to any treatment was given and evaluate their impact on progression-free survival (PFS) and overall survival (OS).

**Patients and methods:**

Forty-seven patients with primary HGSC and ascites were included. Flow-cytometric analysis was performed to detect percentages of CD3^+^ T cells (CD4^+^, CD8^+^, Tregs, and NKT cells), B cells, NK cells (CD56^bright^CD16^−^ and CD56^dim^CD16^+^ subsets), macrophages and dendritic cells (DCs). Furthermore, CD103 expression was analyzed on T cells and their subsets, while PD-1 and PD-L1 expression on all ICs. Cut-off of low and high percentages of ICs was determined by the median of variables, and correlation with PFS and OS was calculated.

**Results:**

CD3^+^ cells were the predominant ICs (median 51%), while the presence of other ICs was much lower (median ≤10%). CD103^+^ expression was mostly present on CD8^+^, and not CD4^+^ cells. PD-1 was mainly expressed on CD3^+^ T cells (median 20%), lower expression was observed on other ICs (median ≤10%). PD-L1 expression was not detected. High percentages of CD103^+^CD3^+^ T cells, PD-1^+^ Tregs, CD56^bright^CD16^−^ NK cells, and DCs correlated with prolonged PFS and OS, while high percentages of CD8^+^ cells, macrophages, and PD-1^+^CD56^bright^CD16^−^ NK cells, along with low percentages of CD4^+^ cells, correlated with better OS only. DCs were the only independent prognostic marker among all ICs.

**Conclusions:**

Our results highlight the potential of ascites tumor-immune microenvironment to provide additional prognostic information for HGSC patients. However, a larger patient cohort and longer follow-up are needed to confirm our findings.

## Introduction

Ovarian carcinoma is a gynecological malignancy with the highest mortality rate in Western countries and the sixth leading cause of cancer-related deaths among women.^1^ High-grade serous carcinoma (HGSC) is the most common and aggressive histological type. Lack of symptoms and adequate screening methods usually result in delayed diagnosis and advanced stage with less than 40% of a 5-year survival rate for HGSC patients.^2^ Cytoreductive surgery combined with carboplatin/paclitaxel chemotherapy (with or without bevacizumab) is still the standard treatment approach.^3^ Despite the good overall response, 70% of the patients experience relapse or develop metastatic disease and resistance. Unfortunately, no significant improvement has been achieved in the last three decades, except for *BRCA1/2* mutated tumors where poly-ADP-ribose polymerase (PARP) inhibitors slightly shifted the care paradigm for ovarian carcinoma.^4^ However, accumulating evidence is showing that tumor-immune microenvironment (TME) in ovarian carcinoma can open the door for the discovery of new prognostic markers and the development of immunotherapeutic treatment approaches. For instance, the presence of tumor-infiltrating CD3^+^ T cells in the primary tumor positively correlates with progression-free survival (PFS) and overall survival (OS) of ovarian carcinoma patients, as does high CD8/CD4 ratio.^5^ Strong association with better OS was also seen on CD3^+^ T cells expressing CD103 tissue resident marker,^6^ while infiltration of regulatory T cells has an opposite impact, and the contribution of B cells remains undefined.^7^ Cells from innate immunity, such as natural killer (NK) cells, dendritic cells (DCs), and macrophages contribute to improved outcomes, except for macrophage subsets that polarize from tumor-inhibiting (M1) to tumor-promoting (M2) phenotype and are associated with disease progression.^8^ Moreover, increased expression of PD-1 and PD-L1 is one of the inhibition mechanisms of anti-tumor response by induction of peripheral tolerance, and TME has a significant role in its activation.^9^ Several studies have examined the feasibility of using PD-1 and PD-L1 to serve as prognostic biomarkers for ovarian carcinoma, although their role is still controversial.

Ascites is the most common sign of advanced ovarian carcinoma. Over the last years, studies have demonstrated that ascites contains almost the same immune cells (ICs) and extracellular components as the primary tumor.^2,10,11^ However, there is a lack of quantitative data about the percentages of ICs in ascites and data on their clinical importance. Moreover, the role of immune checkpoints is also poorly described. Our objective was to quantitate ICs in HGSC ascites at disease presentation, assess the expression of PD-1 and PD-L1 on ICs, and investigate their prognostic significance for PFS and OS.

## Patients and methods

### Patients

Patients diagnosed with primary HGSC between January 2019 and May 2021 at the Institute of Oncology Ljubljana (IOL) and/or University Medical Centre Ljubljana were included in the study. The inclusion criteria were as follows: age > 18 years, WHO performance status from 0–1, histologically confirmed HGSC, International Federation of Gynecology and Obstetrics (FIGO) stage ≥ IIIB, presence of malignant ascites, and indication for first-line systemic treatment with platinum agents. All patients received standard chemotherapy treatment. The study was approved by the National Ethics Committee in Ljubljana, Slovenia (0120-33/303/2018/3 and 0120-33/303/2018/6). All patients signed informed consent before inclusion in the study. The study was conducted in accordance with the Helsinki Declaration and Good Clinical Practice.

### Study design

Ascites samples were collected at disease presentation, specifically during laparoscopy or laparotomy before the tumor biopsy was performed and any treatment was initiated, and were immediately sent to the Department of Cytopathology, IOL, where were processed as previously described by our group.^12^ Aliquots of ascites were prepared for flow-cytometric analysis. Percentages of T cells, B cells, NK cells, macrophages, and DCs, and expression of CD103, PD-1, and PD-L1 were analyzed. Their correlation with patient's PFS and OS was calculated. Survival analysis was based on a 3-year patient follow-up. Clinical data were obtained from patient's electronic medical record. Treatment characteristics such as type of surgery, residual disease after surgery, chemotherapy, treatment with bevacizumab or olaparib, and quantity of ICs in the ascites were also analyzed and further correlated with PFS and OS. Surgery was defined as primary (first treatment procedure), secondary (interval surgery after neoadjuvant chemotherapy was possible), or no surgery (interval surgery after neoadjuvant therapy was not possible because the tumor was still inoperable). Residual disease after primary or interval surgery was defined as no residual tumor, residual tumor ≤ 1 cm, or residual tumor > 1 cm. Chemotherapy was defined neoadjuvant (before surgery), or adjuvant (after surgery).

### Flow-cytometric analysis

**FIGURE 1.**
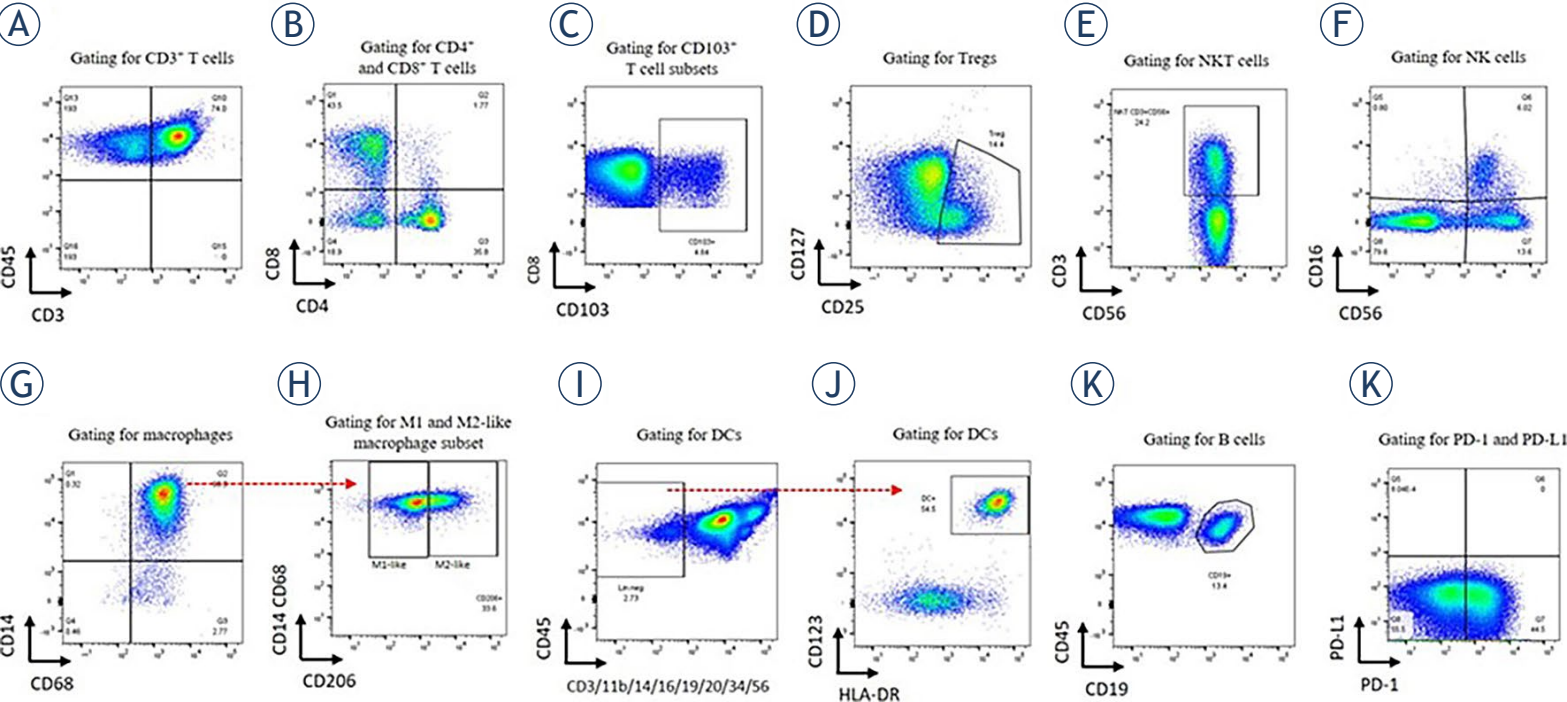
Gating strategy for immune cells in ascites. **(A)** CD3^+^ T cells were gated on CD45^+^. **(B)** The depicted gate shows CD4^+^ *vs*. CD8^+^ subsets gated on CD3^+^ T cells and **(C)** CD103 expression on CD8^+^. The same gating strategy was applied for CD103 expression on CD4^+^ (not shown). **(D)** Gating representative for Tregs. The dot plot depicts CD4^+^ cells discriminated according to CD127 and CD25 positivity. **(E)** NKT cells were gated according to CD3 and CD56 positivity. **(F)** NK cells were gated according to CD16 and CD56 positivity. Two subsets were defined: CD56^bright^CD16^−^ and CD56^dim^CD16^+^. **(G)** The dot plot depicts macrophages according to CD14 and CD68 positivity (pre-gated on CD11b^+^CD45^+^ cells). **(H)** M1-like macrophages were defined as CD206^−^ macrophages, and M2-like as CD206^+^ macrophages. **(I)** DCs were gated per exclusion − as lineage-negative cells (no expression of CD3/CD11b/CD14/CD16/CD19/CD20/CD34/CD56) and further discriminated by CD123 and HLA-DR positivity. **(J)** B cells were gated as CD19^+^ cells per CD45^+^. **(K)** PD-1 and PD-L1 positivity was detected on each cell population/subset.

Sample preparation for flow-cytometric measurement was carried out as previously described by our group.^13^ Antibodies (Supplementary Table 1) were divided into 5 test tubes according to the analyzed ICs (Supplementary Figure 1) and half a million cells per 100 μl were put in each tube. Flow-cytometric data was acquired with a 10-color BD FACSCanto™ II Flow Cytometer and FACSDiva 8.0.2 software (BD Bioscience, USA). FSC files were analyzed using FlowJo v10.8 1 (BD Biosciences, USA). Different ICs were gated according to their immunophenotype (Figure 1): T cells (CD3^+^), helper T cell subset (CD4^+^), cytotoxic T cell subset (CD8^+^), regulatory T cell subset (Tregs; CD4^+^CD25^+^CD127^±^), NKT cell subset (CD3^+^CD56^+^), B cells (CD19^+^), NK cells (CD3^−^CD56^dim^CD16^+^ and CD3^−^CD56^bright^CD16^−^ subsets), macrophages (CD11b^+^CD14^+^CD68^+^) and their M1-like (CD206^−^) and M2-like (CD206^+^) subsets, and DCs (lineage^−^ (CD3^−^CD11b^−^CD14^−^CD16^−^CD19^−^CD20^−^CD34^−^CD56^−^)HLADR^+^CD123^+^CD11c^+^). Expression of CD103 was analyzed on CD3^+^, CD4^+^, and CD8^+^ T cells. Percentages of T cells, B cells, NK cells, macrophages, and DCs were given as a ratio per CD45^+^. Percentages of CD4^+^, CD8^+^, Tregs, and NKT cells were given as a percentage of CD3^+^, while M1-like and M2-like macrophages were given as a percentage per all macrophages. Expression of PD-1 and PD-L1 was analyzed on each IC population/subset separately.

### Statistical analysis

Descriptive statistics was used to describe the basic features of the data. The median (range) was calculated for each IC population/subset. Mann–Whitney U and Kruskal-Wallis nonparametric tests were used to compare if there were differences in the percentages of ICs, and PD-1 and PD-L1 expression levels among IC subsets and within different treatment characteristics. A cut-off value of low and high percentages of ICs was determined by the median of the variables. Kaplan Maier method (with log-rank test) was used to evaluate PFS and OS for treatment characteristics, as well as PFS and OS for low and high percentages of ICs. PFS was calculated as the time from diagnosis until disease progression or death, and OS was calculated as the time from diagnosis to death. Hazard ratio (HR) and 95% confidence interval (CI) were calculated for both univariate and multivariate analysis. Parameters that proved to be significant in the univariate analysis were included in the multivariate analysis. Median survival was expressed in months. P < 0.05 was considered significant. Statistical analysis was performed with IBM SPSS v 28.0.1.0 (142) and GraphPad Prism 9 statistic software.

## Results

### Patients and treatment characteristics

Forty-seven patients with histologically confirmed HGSC and ascites were included in the study. Ascites was collected at disease presentation and prior to any treatment. The mean age of the patients was 64 years (range 41–84 years). Eleven patients underwent primary surgery, resulting in no residual tumor after surgery in 6/11 patients and residual tumor ≤ 1 cm in 5/11 patients. All 11 patients were then treated with adjuvant chemotherapy. Twenty-three patients underwent neoadjuvant chemotherapy followed by interval surgery and adjuvant chemotherapy. No residual tumor was achieved in 13/23 patients, while residual tumor ≤ 1 cm and residual tumor > 1 cm were present in 7/23 and 3/23 patients, respectively. The remaining 13 patients were inoperable and were treated only with chemotherapy. Furthermore, 16/47 patients were post-surgery treated with bevacizumab maintenance for 15 months or until disease progression, while 12/47 patients with known *BRCA1/2* mutation with olaparib maintenance for 24 months or until disease progression. In our 3-year follow-up analysis, 36/47 patients had disease progression, and 23/47 patients died (Table 1).

**TABLE 1.** Clinical characteristics of the patients included in the study

**Age at diagnosis (years)**	
**Mean**	64
**Range**	41–84
**FIGO stage (N, %)**	
**IIIB**	1 (2)
**IIIC**	34 (72)
**IVA**	7 (15)
**IVB**	5 (11)
**Surgery (N, %)**	
**Primary**	11 (23)
**Interval**	23 (49)
**No surgery (remained inoperable)**	13 (28)
**Residual disease after surgery (N, %)**	
**No residual tumor**	19 (40)
**Residual tumor ≤ 1 cm**	12 (26)
**Residual tumor > 1 cm**	3 (6)
**Chemotherapy (N, %)**	
**Adjuvant**	11 (23)
Neoadjuvant*	36 (77)
**Bevacizumab (N, %)**	
**No**	31 (66)
**Yes**	16 (34)
**Positive family history (N, %)**	
**No**	30 (64)
**Yes**	17 (36)
***BRCA1/2* mutation (N, %)**	
**No**	31 (66)
**Yes**	12 (26)
**Unknown**	4 (9)
**Olaparib (N, %)**	
**No**	35 (74)
**Yes**	12 (26)
**Disease progression**	
**No**	11 (23)
**Yes**	36 (47)
**Death**	
**No**	24 (51)
**Yes**	23 (49)

* 13 of these patients were inoperable and received only chemotherapy, while the other 23 patients were operable and received adjuvant chemotherapy as well

**FIGURE 2.**
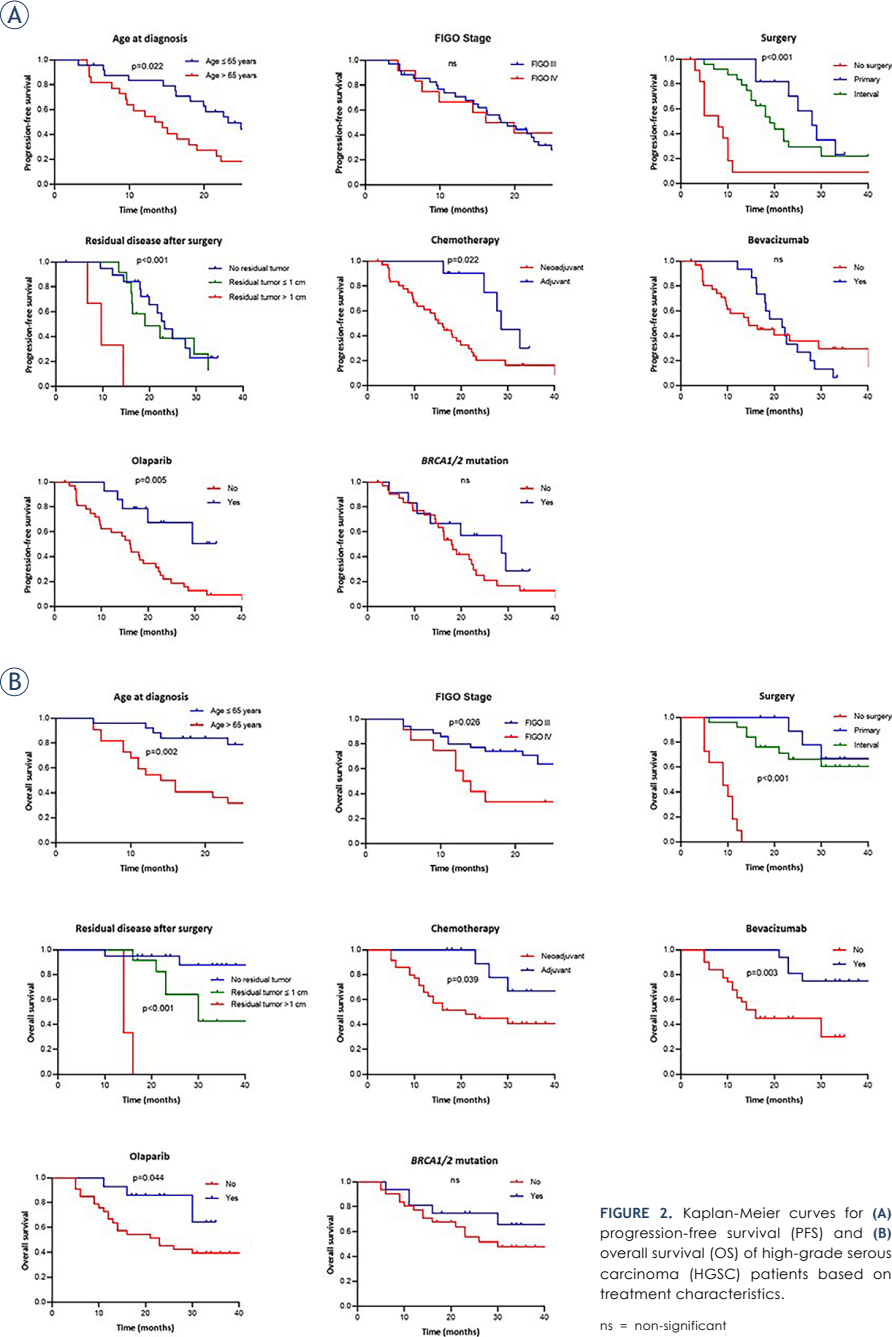
Kaplan-Meier curves for **(A)** progression-free survival (PFS) and **(B)** overall survival (OS) of high-grade serous carcinoma (HGSC) patients based on treatment characteristics. ns = non-significant

Patients, diagnosed with HGSC at age ≤ 65 years had significantly better PFS and OS than older patients (PFS: p = *0.022*, 22.6 vs. 13.1 months; OS: p = *0.002,* 74.2 vs. 54.8 months). As expected, patients diagnosed at FIGO stage III demonstrated significantly better OS outcomes when compared to patients diagnosed at FIGO stage IV (p = 0.026, 23.0 *vs*. 13.0 months). Furthermore, patients who underwent surgery (primary or interval) and adjuvant chemotherapy had significantly better PFS and OS than those with no surgery and neoadjuvant chemotherapy (PFS: p < 0.001 and p = 0.022; OS: p < 0.001 and p = 0.039, respectively). There was no difference in PFS and OS among the patients who underwent primary surgery and those who underwent interval surgery. Treatment with bevacizumab did not affect PFS (p = 0.950, median 20.4 *vs*. 14.2 months), but OS was prolonged (p = 0.003, median 33 *vs*. 16 months). Treatment with olaparib for patients with known *BRCA1/2* mutation indicated significantly better PFS (p = 0.005, median 19.75 *vs*. 16.13 months) and OS (p = 0.044, median 21.5 *vs*. 23 months) (Table 2, Figure 2).

**TABLE 2.** Univariate analysis of patient's survival based on treatment characteristics

	**Progression-free survival**			**Overall survival**		

	**HR (95% Cl)**		**P-value**	**HR (95% Cl)**		**P-value**
**Age at diagnosis (≤ 65 *vs.* > 65 years)**	0.504	0.266–0.954	**0.022**	0.271	0.117–0.627	**0.002**
**FIGO stage (III *vs.* IV)**	1.331	0.691–2.566	0.381	0.312	0.112–0.872	**0.026**
**Surgery**		**< 0.001**			**< 0.001**	
**no *vs.* primary**	3.365	1.248–9.072	**0.002**	9.907	3.045–32.23	**< 0.001**
**no *vs.* interval**	2.981	1.133–7.845	**0.001**	8.529	2.270–32.05	**< 0.001**
**primary *vs.* interval**	0.570	0.253–1.282	0.192	0.605	0.186–1.973	0.239
**Residual disease after surgery**		< 0.0001			< 0.001	
**no residual tumor *vs.* ≤ 1cm**	0.764	0.314–1.858	0.538	0.189	0.045 –0.799	**0.020**
**no residual tumor *vs.* > 1cm**	0.121	0.006–2.256	**< 0.001**	0.066	0.003–1.408	**< 0.001**
**residual tumor ≤ 1cm *vs.* > 1cm**	0.131	0.008–2.209	**< 0.001**	0.131	0.009–1.988	**< 0.001**
**Chemotherapy (adjuvant *vs.* neoadjuvant)**	0.358	0.177–0.725	**0.022**	0.307	0.129–0.732	**0.039**
**Bevacizumab (no/yes)**	0.979	0.500–1.917	0.950	4.280	1.888–9.703	**0.003**
***BRCA1/2* mutation (no *vs.* yes)**	1.721	0.827–3.584	0.186	1.734	0.698–4.307	0.274
**Olaparib (no *vs.* yes)**	3.486	1.765–6.884	**0.005**	3.148	1.329–7.629	**0.044**

### Immune cells, and PD-1 and PD-L1 expression in the ascites at disease presentation

**FIGURE 3.**
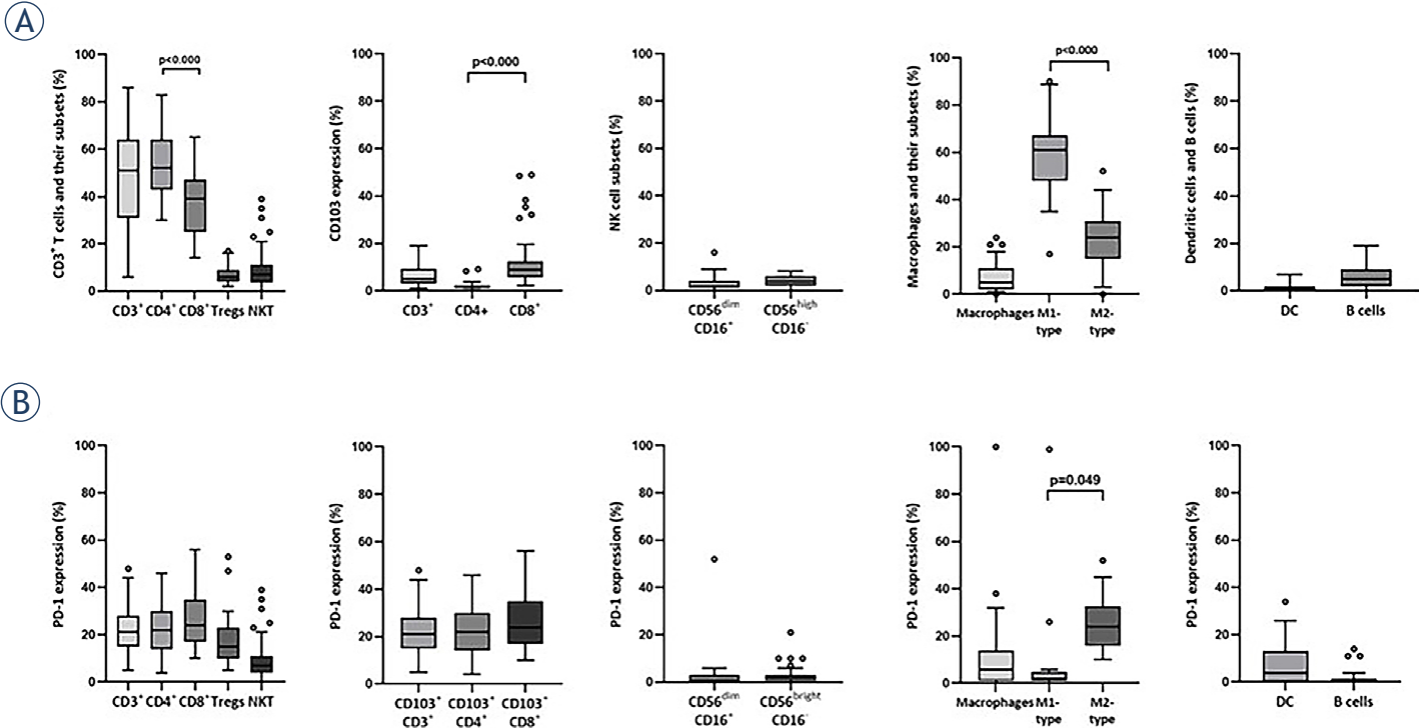
Box plots showing median (range) and quartiles for **(A)** T cells, NK cells, macrophages, DCs, B cells, and their subsets in the ascites of HGSC patients, and **(B)** the expression of PD-1 for each immune population/subset at disease presentation. CD3^+^ T cells, NKT cells, CD56^bright^CD16^−^ and CD56^dim^CD16^+^ NK cells, macrophages, DCs, and B cells are given as a percentage per all CD45^+^ cells, while each subset is given as a percentage per its main population.

CD3^+^, CD4^+^, and CD8^+^ T cells were measured in ascites samples of all 47 patients. Due to the limited number of cells in some samples, Tregs, NKT cells, NK cells, B cells, macrophages, DCs, and CD103 expression were measured in 39 samples, and due to the later inclusion of CD206 antibody in our study, M1-like and M2-like macrophages were analyzed in only 15 samples. PD-1 and PD-L1 expression was measured in 39 out of 47 samples (Supplementary Table 2). The results of ICs (Figure 3 A) showed a predominance of CD3^+^ T cells in ascites, with a median percentage of 51% (range 6–86). In fact, the medians of CD4^+^ subsets and CD8^+^ subsets were 52% (range 30–83%) and 39% (range 14–63%), respectively, with CD4^+^ being significantly more abundant (p < 0.001). The median frequency for Tregs was 6% (range 2–17%). CD103 was expressed on CD3^+^ T cells (median 3%, range 1–34%). The majority of CD3^+^ T cells that expressed CD103^+^ were CD8^+^ (median 9%, range 2–49%), while only a small minority of CD4^+^ showed expression of CD103 (p < 0.001, median 2%, range 1–9%). We also examined the frequency of NKT cells and NK cells. The median percentage of NKT cells was 7% (range 1–39%) and of NK cells 6% (range 1–16). More precisely, 2% (range 1–16) of the NK cells were CD56^dim^CD16^+^, and 4% (range 1–8) were CD56^bright^CD16^−^. The median percentage of macrophages was 5% (1–24%), 61% of them were M1-type (range 17–90%), and 24% (range 1–52%) were M2-type. M1-like macrophages were significantly more abundant than M2-like macrophages (p < 0.001). We also identified the presence of DCs with a median frequency of 1% (range 1–7%) and B cells with 5% (range 1–19%). Furthermore, PD-1 was mainly expressed on T cells, without significant differences among CD4^+^, CD8^+^, and Treg subsets. The median expression for all T cell subsets was roughly 20%. Similar results were found for CD103^+^ positive T cells. Macrophages and DCs had slightly lower PD-1 expression than T cells (median < 10%). Significantly higher PD-1 expression was observed on M2-like macrophages (median 24%, range 1–52%) compared to M1-like macrophages (p = 0.049; median 1%, range < 1–26%). NK cells and B cells had the lowest expression of PD-1 (median < 2%) (Figure 3 B). We did not detect an expression of PD-L1 in any of the analyzed ICs.

### Immune cells and their association with treatment characteristics

**FIGURE 4.**
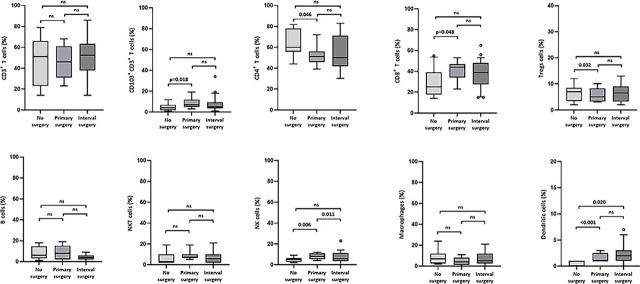
Box plots showing the median (range) and quartiles for the percentages of different immune cells at presentation and their association with surgery type (no surgery *vs*. primary *vs*. interval) the patients were later assigned with.

**FIGURE 5.**
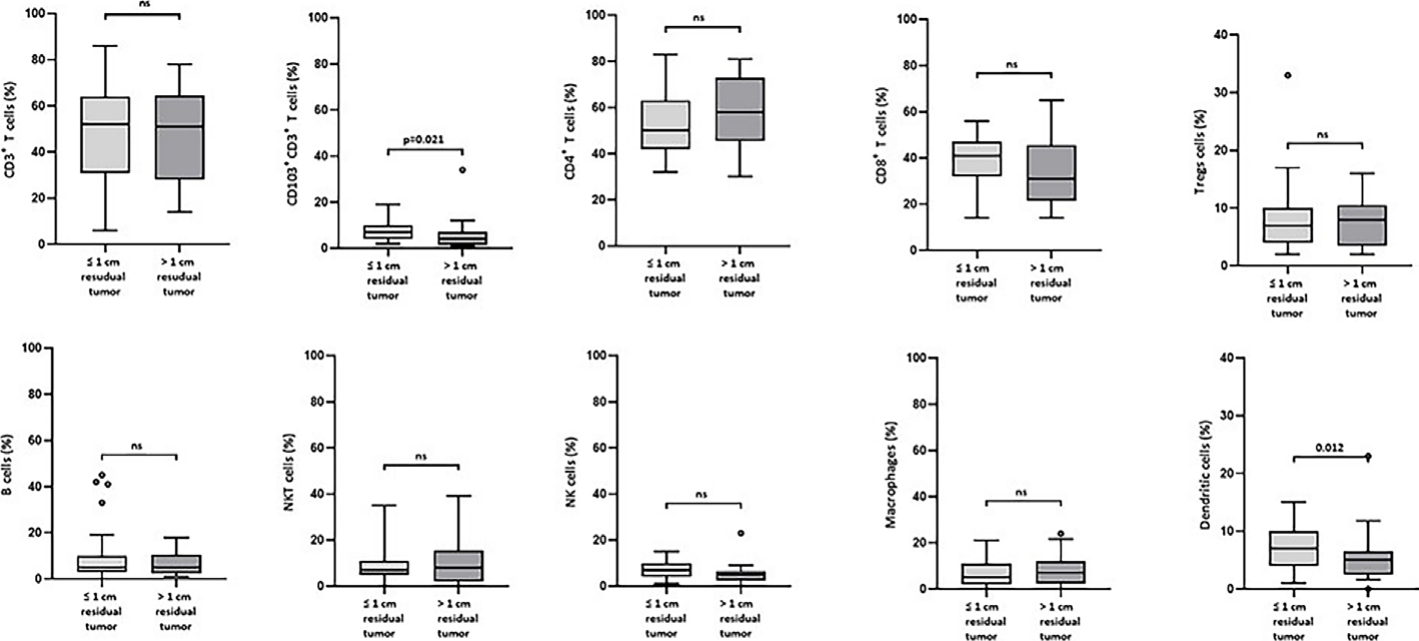
Box plots showing the median (range) and quartiles for the percentages of different immune cells at presentation and their association with residual disease after surgery (less (no and ≤ 1cm) residual tumor *vs*. more (< 1 cm and inoperable) residual tumor).

Furthermore, we aimed to determine if there are differences in the percentages of ICs at disease presentation that could be associated with treatment characteristics of HGSC such as primary operability (ability to perform primary surgery) and residual disease after surgery. According to the Kruskal-Wallis overall comparison, significant differences among the three surgery subgroups (no surgery, primary, and interval surgery) were observed for NK cells (p = 0.014) and DCs (p = 0.003) with lower percentages of NK cells and DCs in inoperable patients. However, according to the pairwise comparison within the subgroups, in addition to the association with lower NK cells (p = 0.006) and DCs (p = 0.001), lower percentages of CD103^+^CD3^+^ T cells (p = 0.018), CD8^+^ (p = 0.048), and higher percentages of CD4^+^ (p = 0.046) and Treg (p = 0.032) were observed in the inoperable patient group *vs*. primary surgery group (Figure 4). When we compared the residual disease with percentages of ICs we observed an association with significantly lower percentages of CD103^+^ CD3^+^ T cells and DCs in the patients with more residual tumor (Figure 5).

**FIGURE 6.**
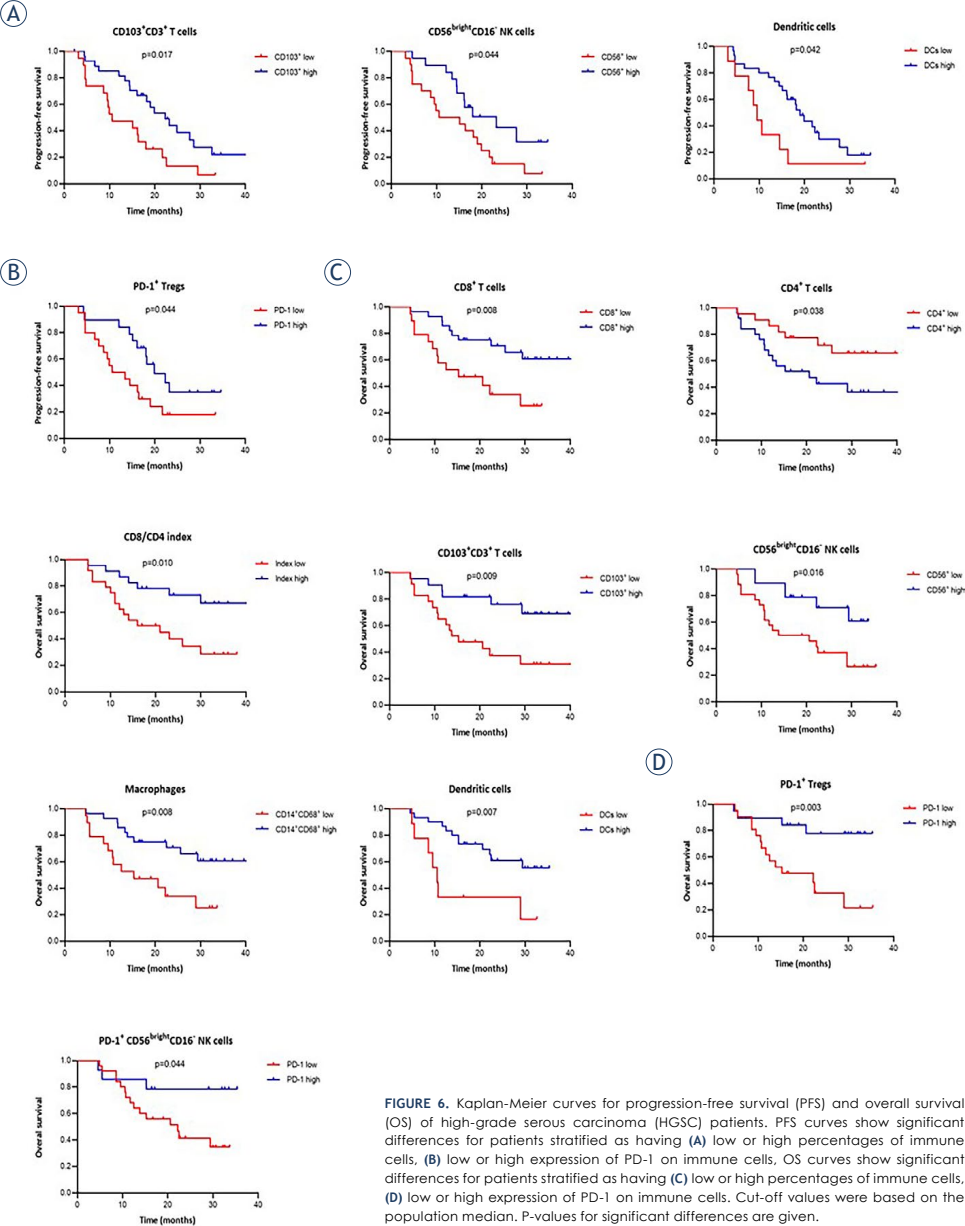
Kaplan-Meier curves for progression-free survival (PFS) and overall survival (OS) of high-grade serous carcinoma (HGSC) patients. PFS curves show significant differences for patients stratified as having **(A)** low or high percentages of immune cells, **(B)** low or high expression of PD-1 on immune cells, OS curves show significant differences for patients stratified as having **(C)** low or high percentages of immune cells, **(D)** low or high expression of PD-1 on immune cells. Cut-off values were based on the population median. P-values for significant differences are given.

We also wanted to see if high and low percentages of ICs correlate with PFS and OS. Patients stratified by having high percentages of CD103^+^CD3^+^ T cells (p = 0.017, median 18.6 *vs*. 10.6 months), CD56^bright^CD16^−^ NK cells (p = 0.044, median 17.2 *vs*. 12.8 months) and DCs (p = 0.042, median 17.8 *vs*. 9.5 months) were associated with significantly better PFS compared to patients having low percentages of these ICs (Table 3, Figure 5 A). Also, a trend towards longer PFS was observed in patients stratified by having low percentages of CD4^+^, high percentages of CD8^+^, and all macrophages (Table 2, Supplementary Figure 2 A). For Tregs, B cells, M1-like and M2-like macrophage subsets, CD56^dim^CD16^+^ NK subsets, and CD8/CD4 index, no differences in PFS for patients stratified by having high and low percentages were seen. We also observed a significant association with better PFS for patients with high PD-1 expression on Tregs (p = 0.044, median 18.2 *vs*. 12.0 months) (Figure 5 B), and a trend towards better PFS for patients stratified by having a low expression of PD-1 on NKT (median 17.8 *vs*. 15.1 months) and high expression of PD-1 on M2-like macrophages (median 17.2 *vs*. 14.2 months) (Supplementary Figure 1 B). PD-1 expression on the other ICs showed no difference between patients with low and high PD-1 expression rates in the ascites.

**TABLE 3.** Univariate analysis of patient's survival based on the low/high percentages of immune cells in the ascites at disease presentation

	**Progression-free survival**			**Overall survival**		

	**HR (95% Cl)**		**P-value**	**HR (95% Cl)**		**P-value**
**CD3^+^ (low *vs*. high)**	1.098	0.563–2.142	0.777	0.670	0.301–1.493	0.324
**CD4^+^ (low *vs*. high)**	0.547	0.260–1.150	0.110	0.401	0.174–0.928	**0.038**
**CD8^+^ (low *vs*. high)**	1.918	0.887–4.148	0.066	2.854	1.182–6.889	**0.008**
**CD8/CD4 index (low *vs*. high)**	1.076	0.560–2.069	0.820	2.973	1.304–6.780	**0.010**
**Tregs (low *vs*. high)**	0.856	0.412–1.778	0.677	0.807	0.324–2.006	0.649
**CD103^+^CD3^+^ (low *vs*. high)**	2.152	1.050–4.408	**0.017**	3.234	1.365–7.661	**0.009**
**CD103^+^CD4^+^ (low *vs*. high)**	0.515	0.232–1.147	0.108	0.816	0.328–2.031	0.661
**CD103^+^CD8^+^ (low *vs*. high)**	1.984	0.899–4.379	0.085	1.744	0.706–4.309	0.245
**NKT cells (low *vs*. high)**	0.520	0.246–1.101	0.060	1.208	0.507–2.878	0.661
**CD56^bright^CD16^−^ NK cells (low *vs*. high)**	2.111	1.013–4.396	**0.044**	2.903	1.304–6.464	**0.016**
**CD56^dim^16^+^ NK cells (low *vs*. high)**	1.399	0.694–2.820	0.362	1.851	0.756–4.533	0.157
**Macrophages (low *vs*. high)**	0.601	0.245–1.478	0.275	1.943	0.754–5.006	**0.008**
**M1-like macrophages (low *vs*. high)**	0.868	0.224–3.370	0.835	1.562	0.321–7.604	0.533
**M2-like macrophages (low *vs*. high)**	2.142	0.553–8.297	0.224	2.854	1.182–6.889	0.080
**B cells (low *vs*. high)**	1.161	0.560–2.450	0.686	0.464	0.188–1.141	0.102
**DCs (low *vs*. high)**	2.245	0.799–6.310	**0.042**	3.307	0.939–11.65	**0.007**

We observed significantly longer OS in patients stratified by having high percentages of CD103^+^CD3^+^ T cells (p = 0.009, median 22.7 *vs*. 15.8 months), CD8^+^ T cells (p = 0.008, median 27.3 *vs*. 15.3 months), CD56^bright^CD16^−^ NK cells (p = 0.016, median 22.2 *vs*. 17.2 months), macrophages (p = 0.008, median 22.7 *vs*. 17.7 months) and DCs (p = 0.007, median 26.5 *vs*. 16.4 months), and low percentages of CD4^+^ T cells (p = 0.038, median 27.3 *vs*. 16.5 months) compared to their counterparts. Furthermore, patients with high CD8/CD4 index were associated with significantly longer OS compared to patients with low CD8/CD4 index (p = 0.010, median 30.0 *vs*. 16.5 months) (Table 3, Figure 4 C). For other ICs, no significant correlations with OS were observed. Furthermore, high expression of PD-1 on Tregs (p = 0.003, median 29.4 *vs*. 14.6 months) and CD56^bright^CD16^−^ cells (p = 0.044, median 22.2 *vs*. 10.6 months) showed significantly better OS (Table 3, Figure 5 D). In addition, a trend towards better OS was seen for patients stratified by having low PD-1 expression on NKT and high PD-1 expression on CD56^dim^CD16^+^ NK cells and B cells (Supplementary Figure 3 B).

We also performed a multivariate analysis of significant parameters among treatment characteristics and IC populations affecting patient's survival. Considering the low number of patients and presence of multiple subgroups in the clinical parameters, multivariant analysis required re-categorizing surgery type as either no *vs*. primary surgery, and residual disease after surgery as either no and ≤ 1 cm of residual tumor *vs*. residual tumor > 1 cm and inoperable tumor, and no more than five significant variables were chosen. According to the results of multivariate analysis, only residual tumor after surgery was identified as an independent prognostic marker for both PFS (p = 0.046) and OS (p < 0.001) among treatment characteristics and DCs (low *vs*. high) as an independent prognostic marker among ICs for PFS (p = 0.049) only (Table 4).

**TABLE 4.** Multivariate analysis of the treatment characteristics and immune cells

**Variables included in the multivariate analysis**	**Progression-free survival**			**Overall survival**		

	**HR (95% Cl)**		**P-value**	**HR (95% Cl)**		**P-value**
**Primary surgery (no *vs*. yes)**	0.640	0.194–2.114	0.509	0.592	0.101–3.454	0.560
**Residual disease after surgery (no residual tumor and ≤ 1 cm of residual tumor *vs*. > 1 cm residual tumor)**	0.408	0.169–0.983	**0.046**	0.009	0.001–0.092	**< 0.001**
**CD103^+^CD3^+^ (low *vs*. high)**	0.605	0.266–1.374	0.230	0.632	0.182–1.307	0.470
**CD56^bright^CD16^−^ NK cells (low *vs*. high)**	1.707	0.683–4.265	0.252	NA	NA	NA
**DCs (no *vs*. yes)**	0.394	0.155–0.998	**0.049**	0.419	0.135–1.307	0.134
**Macrophages (low *vs*. high)**	NA	NA	NA	0.592	0.101–3.545	0.560

NA = no available

## Discussion

HGSC is the most aggressive gynecological malignancy which is usually diagnosed at advanced stages when the disease has already spread in the peritoneum.^14^ Ascites is therefore often the first sign of the disease.^15^ We hypothesized that ICs in ascites might be a promising source of novel prognostic markers for HGSC. We assessed the presence of different ICs together with CD103, PD-1, and PD-L1 expression levels and showed that percentages of cytotoxic ICs (CD8^+^, CD56^bright^CD16^−^ NK cells) as well as macrophages, might affect patient's survival. We also showed that DCs are independent prognostic marker for PFS of HGSC patients.

As expected, our results on clinical and treatment characteristics of HGSC patients included in the study aligned with the already published data on the impact of age at diagnosis, FIGO stage, surgery, residual disease, and chemotherapy and maintenance therapy.^2,3,4^ This data confirms the adequacy of our analyzed patient cohort.

According to the evaluation of ICs in ascites, our findings demonstrated that CD3^+^ T cells (median 51%) are the predominant population in the ascites of HGSC patients (FIGO stage ≥ III, ascites collected before initiation of treatment), with CD4^+^ significantly more abundant than CD8^+^, while the amount of Tregs was low as well as the other ICs investigated. Auer *et al*. reported higher percentages of CD3^+^ (median 80%) compared to our results, with an equal ratio of CD4^+^ and CD8^+^ subsets, and higher percentages of NKT cells (median 15%).^1^ They did not specify patient's FIGO stage and when ascites was collected, which could explain the difference in the results of our and their study. However, the percentages of Tregs, NK cells, and B cells in their study were in concordance with our findings.^1^ Similar percentages of CD4^+^ and Tregs, T cells, and DCs as in our study were reported in other studies.^2,16^ We detected low percentages of macrophages (median 5%) in our series of ascites samples; the majority of them were M1-like. On the contrary, Steitz *et al.* described roughly 70% of macrophages, with equal ratios of both M1-like and M2-like subsets. Unfortunately, they did not describe disease progression by FIGO stage and ascites collection time either.^17^ Therefore, we speculate that lower percentages of macrophages in our study might be related to the inclusion of ascites samples from HGSC patients at the time of diagnosis, and not later at disease progression.

In the literature, we have found some data about percentages of ICs in HGSC ascites, but to our knowledge, there was no data providing information about the association of ICs at disease presentation and treatment assigned to the patients. Interestingly, in our study, we observed higher percentages of CD103^+^, CD8^+^, Tregs, NK cells, and DCs, and lower percentages of CD4^+^ cells in the ascites of patients with less tumor burden that underwent primary surgery compared to inoperable patients, which due to the size of the tumor were no eligible for surgery. These results might indicate an association of the amount and cell type of ICs in ascites at disease presentation with the extent of the tumor burden. Furthermore, we observed higher percentages of CD103^+^ T cells and DCs in patients who underwent interval surgery compared to the inoperable group. This data suggests the possibility of using these ICs to help us predict which patients, after receiving neoadjuvant chemotherapy, are likely to be eligible for surgery later on and have a lower amount of residual tumor. However, a much larger patient cohort is needed to confirm these findings. And as mentioned above, we have not found any similar studies to compare our results with.

Furthermore, most of the research on the influence of ICs on HGSC patient's survival is carried out on primary tumor tissues, and very little is known about the role of ICs in ascites. For instance, it has been reported that T cells in primary tumors improve the survival of HGSC patients. In fact, CD8^+^ cells correlated with improved survival, and Tregs, as well as CD4^+^ cells were seen as an indicator of poor prognosis.^18,19^ On the other hand, studies on T cells in ascites have failed to confirm this correlation, even though a trend towards improved survival in patients with low CD4^+^ T cells was reported.^2^ However, the ratio between CD8^+^ and CD4^+^ T cells or even Tregs has been reported as a more appropriate indicator of better OS.^20,21,22,23^ In our study, similarly, we observed an association with significantly longer OS for patients stratified by having low CD4^+^ and high CD8^+^ T cells, and high CD8/CD4 index compared with the patients stratified by having high CD4^+^ and low CD8^+^ T cells, and also a low CD8/CD4 index. Regarding NKT cells, data on their role in ovarian carcinoma survival is generally limited. According to our results, there was not a significant correlation between NKT cells and survival rate for patients stratified as having high percentages of NKT cells. We also investigated the role of CD103 on T cells in HGSC ascites. CD103 is a subunit of the αE/β7 integrin that helps to retain expressing cells on the epithelium.^24^ CD103 has been proposed as a marker of activated and tumor-reactive CD8^+^ T cells in ascites HGSC^25,26^ but no data correlated with survival was given. We showed that CD103 was mostly expressed on CD8^+^, and not on CD4^+^ subsets, which was shown by us and by two other studies.^26,27^ Furthermore, patients stratified by having high percentages of CD103^+^CD3^+^ T cells in HGSC ascites were associated with better PFS and OS. CD103^+^CD3^+^ T cells in ascites seem to have the same potential of prognostic information as reported for the CD103^+^ tumor-infiltrated T cells in the primary tumor^27^ and we speculate that these cells in the ascites might be involved in the improvement of the antitumor response in the peritoneum. Of course, a larger patient cohort and additional tests are needed to gain a more comprehensive understanding of the significance and role of CD103^+^CD3^+^ T cells in HGSC ascites.

The role of B cells regarding their contribution to impaired antitumor immunity in HGSC has not been investigated as much as the role of T cells. However, there are few reports showing a trend towards worse OS in patients with high infiltration of B cells in ascites. These findings are consistent with ours.^28,29^ Interestingly, opposite findings have been reported in primary tumors where a high percentage of B cells correlated with favorable survival, indicating that more studies are needed to estimate the role of B cells in ovarian tumors.^30,31^

DCs in ascites have been poorly investigated. Only one study described a trend of high percentages of DCs in HGSC ascites toward a better survival outcome.^2^ Similarly, we confirmed a significant association between DCs and patient's survival. Consistently, patients stratified by having high percentages of DCs were associated with improved prognosis in the primary tumors as well.^31,32^

NK cells have attracted attention due to their ability to kill tumor cells without prior sensitization. There is limited data on the contribution of NK cell immunity to the clinical outcome of ovarian carcinoma. Infiltration of NK cells in primary tumors has shown a contradictory impact on survival outcomes in HGSC.^33^ However, recently, one study showed an association of high percentages of CD56^+^ NK cells in ascites with better PFS and OS.^14^ Similarly, we showed the same association of both CD56^bright^CD16^−^ and CD56^dim^CD16^+^ NK cells with the survival outcome in our patient cohort. It is generally thought that CD56^bright^CD16^−^ NK cells have a higher capacity for cytokine production and have mainly proliferative potential, and on the contrary, CD56^dim^CD16^+^ NK cells have weak cytotoxic activity,^34,35^ which explains why we observed significant results for CD56^bright^CD16^−^ and only a trend towards CD56^dim^CD16^+^.

Macrophages in ovarian ascites are gaining a lot of attention in recent years, due to their plasticity to switch from antitumor M1 to protumor M2 phenotypes.^8^ M2-type macrophages have been characterized by the expression of markers such as the scavenger receptors CD206 or CD163.^36^ Published data is speculating that M2-type macrophages are taking the main role in immune suppression and angiogenesis promotion to sustain tumor growth.^37,38^ Even though we identified lower percentages of macrophages than reported, we showed that patients stratified by having higher percentages of M1-like macrophages than M2-like macrophages were associated with better survival. We speculate that when the diagnosis is given, even though the total macrophage count is low, M1-like macrophages are predominant and are probably the ones contributing to a better outcome. However, during disease progression, M2-like macrophages outnumbered the M1 subset and most probably contributed to tumor progression and poor outcome of the disease.^39^

Immune tolerance is defined by the inability of ICs to express immune checkpoints such as PD-1 and PD-L1. PD-1 receptor is an inhibitor of both adaptive and innate immune responses and can be expressed on CD8^+^ T cells, CD4^+^ T cells, and Tregs in ovarian tumors, whereas PD-L1 is expressed on activated T cells, tumor-infiltrating macrophages or fibroblasts, contributing to tumor immune escape.^40^ However, the expression of PD-1 and PD-L1 in HGSC ascites and its correlation with survival has not yet been fully investigated. In the present study, we showed that PD-1 expression is present in almost all ICs, except on NK cells and B cells (less than 1%). We detected roughly 20% PD-1 expression on CD4^+^, CD8^+^ T cells and Tregs. However, Imai *et al*. reported 2x higher level of PD-1^+^CD4^+^ and PD-1^+^CD8^+^ cells in the ascites.41 Possible reasons for this discrepancy might be the different PD-1 clone selections for the analysis, as well as the diversity of the patient cohort. Imai *et al*. performed the analysis on different types of malignant epithelial ovarian carcinomas, and a few cases on borderline and benign tumors. Similar to us, they did not find a correlation between survival and PD-1 expression on CD4^+^ and CD8^+^ T cells. However, Sato *et al*. found an association between CD8^+^ cells and PD-1 expression in advanced epithelial ovarian carcinoma.^20^ On the contrary, Pawłowska *et al*. demonstrated an association of high percentages of both PD-1^+^CD4^+^ and PD-1^+^CD8^+^ cells in ascites with worse outcomes, indicating a negative regulation of the anticancer immune response and exhaustion of T cells in the ascites.^42^ Results on primary ovarian tumors have also reported a correlation of higher PD-1 expression on T cells with shorter survival and worse prognosis.^43,44^ For the other ICs in ovarian carcinoma ascites, data on PD-1 expression and survival correlation is also missing. We showed that high expression of PD-1 on Tregs and CD56^bright^CD16^−^ NK cells is associated with better survival. We do not know how to interpret the correlation of high PD-1 expression with better instead of worse survival as expected. Additional studies are necessary to clarify if PD-1 expression on Tregs and CD56^bright^CD16^−^ NK cells could be a positive prognostic marker for patient survival. We are also the first to confirm that PD-L1 is not expressed on ICs in HGSC ascites, since we did not find data on PD-L1 expression on ICs in ascites. One study on primary tumors has found that almost 2/3 of the tumors had a low level of PD-L1 expression, mainly on ICs rather than tumor cells, and the expression of PD-L1 was associated with significantly worse prognosis,^45^ indicating location-dependent loss of expression of PD-L1 on ICs in ascites.

Nevertheless, it is worth mentioning that all published data on ovarian carcinoma also reports results on low and high percentages of ICs in ascites in correlation with patient survival without stratifying patients in subgroups according to their treatment characteristics. This is due to the low number of HGSC patients^2,16,26,27,28,32^ each research group confronts, and also the reason why we did not conduct that kind of analysis within the treatment subgroups either. Yet, the multivariate analysis indicated that residual tumor is the only independent prognostic marker for PFS and OS, and DCs are an independent prognostic marker for PFS only. We believe that multicentric studies on large patient cohorts could give more accurate information on the prognostic meaning of DCs and other ICs in the ascites.

In conclusion, we found that CD3^+^ were the predominant cells in HGSC ascites at disease presentation and showed that high levels of CD103^+^CD3^+^ T cells, CD56^bright^CD16^−^ NK cells and DCs improve both PFS and OS, whereas high levels of CD8^+^, CD8/CD4 index, macrophages, PD-1^+^ Tregs and PD-1^+^CD56^bright^CD16^−^ NK cells, and low levels of CD4^+^ improve OS only. We also confirmed that the residual disease is the only clinical independent prognostic marker for PFS and OS, and we showed that DCs are the only ICs that might become an independent prognostic marker for PFS. Data obtained highlight the potential of ascites as a source to provide additional prognostic information for HGSC patients. However, a larger patient cohort and longer follow-up are necessary to assess the independent prognostic significance of ICs together within different treatment characteristics.

## Supplementary Material

Supplementary Material Details

## References

[1] Auer K, Bachmayr-Heyda A, Sukhbaatar N, Aust S, Schmetterer KG, Meier SM (2016). Role of the immune system in the peritoneal tumor spread of high grade serous ovarian cancer. Oncotarget.

[2] Wefers C, Duiveman-de Boer T, Yigit R, Zusterzeel PLM, van Altena AM, Massuger LFAG (2019). Survival of ovarian cancer patients is independent of the presence of DC and T cell subsets in ascites. Front Immunol.

[3] du Bois A, Quinn M, Thigpen T, Vermorken J, Avall-Lundqvist E, Bookman M (2005). 2004 consensus statements on the management of ovarian cancer: final document of the 3rd international gynecologic cancer intergroup ovarian cancer consensus conference (GCIG OCCC 2004). Ann Oncol.

[4] Zhu C, Xu Z, Zhang T, Qian L, Xiao W, Wei H (2021). Updates of pathogenesis, diagnostic and therapeutic perspectives for ovarian clear cell carcinoma. J Cancer.

[5] Zhang L, Conejo-Garcia JR, Katsaros D, Gimotty PA, Massobrio M, Regnani G (2003). Intratumoral T cells, recurrence, and survival in epithelial ovarian cancer. N Engl J Med.

[6] Webb JR, Milne K, Watson P, Deleeuw RJ, Nelson BH (2014). Tumor-infiltrating lymphocytes expressing the tissue resident memory marker CD103 are associated with increased survival in high-grade serous ovarian cancer. Clin Cancer Res.

[7] Gupta P, Chen C, Chaluvally-Raghavan P, Pradeep SB (2019). Cells as an immune-regulatory signature in ovarian cancer. Cancers.

[8] Gupta V, Yull F, Khabele D (2018). Bipolar tumor-associated macrophages in ovarian cancer as targets for therapy. Cancers.

[9] Piętak P, Pietrzyk N, Pawłowska A, Suszczyk D, Bednarek W, Kotarski J (2018). The meaning of PD-1/PD-L1 pathway in ovarian cancer pathogenesis. Wiad Lek.

[10] Preston CC, Maurer MJ, Oberg AL, Visscher DW, Kalli KR, Hartmann LC (2013). The ratios of CD8^+^ T cells to CD4^+^CD25^+^ FOXP3^+^ and FOXP3^−^ T cells correlate with poor clinical outcome in human serous ovarian cancer. PLoS One.

[11] Nakano M, Ito M, Tanaka R, Yamaguchi K, Ariyama H, Mitsugi K (2018). PD-1+ TIM-3+ T cells in malignant ascites predict prognosis of gastrointestinal cancer. Cancer Sci.

[12] Miceska S, Škof E, Novaković S, Stegel V, Jeričević A, Grčar Kuzmanov B (2023). Cytopathological assessment is an accurate method for identifying immunophenotypic features and BRCA1/2 mutations of high-grade serous carcinoma from ascites. Cancer Cytopathol.

[13] Brozic A, Pohar Marinsek Z, Novakovic S, Kloboves Prevodnik V (2015). Inconclusive flow cytometric surface light chain results can cytoplasmic light chains, Bcl-2 expression and PCR clonality analysis improve accuracy of cytological diagnoses in B-cell lymphomas?. Diagn Pathol.

[14] Hoogstad-van Evert JS, Bekkers R, Ottevanger N, Jansen JH, Massuger L, Dolstra H (2020). Harnessing natural killer cells for the treatment of ovarian cancer. Gynecol Oncol.

[15] Ford CE, Werner B, Hacker NF, Warton K (2020). The untapped potential of ascites in ovarian cancer research and treatment. Br J Cancer.

[16] Sato S, Matsushita H, Shintani D, Kobayashi Y, Fujieda N, Yabuno A (2022). Association between effector-type regulatory T cells and immune checkpoint expression on CD8^+^ T cells in malignant ascites from epithelial ovarian cancer. BMC Cancer.

[17] Steitz AM, Steffes A, Finkernagel F, Unger A, Sommerfeld L, Jansen JM (2020). Tumor-associated macrophages promote ovarian cancer cell migration by secreting transforming growth factor beta induced (TGFBI) and tenascin C. Cell Death Dis.

[18] Curiel TJ, Cheng P, Mottram P, Alvarez X, Moons L, Evdemon-Hogan M (2004). Dendritic cell subsets differentially regulate angiogenesis in human ovarian cancer. Cancer Res.

[19] Almeida-Nunes DL, Mendes-Frias A, Silvestre R, Dinis-Oliveira RJ, Ricardo S (2022). Immune tumor microenvironment in ovarian cancer ascites. Int J Mol Sci.

[20] Sato E, Olson SH, Ahn J, Bundy B, Nishikawa H, Qian F (2005). Intraepithelial CD8^+^ tumor-infiltrating lymphocytes and a high CD8^+^/regulatory T cell ratio are associated with favorable prognosis in ovarian cancer. Proc Natl Acad Sci U S A.

[21] Ning F, Cole CB, Annunziata CM (2021). Driving immune responses in the ovarian tumor microenvironment. Front Oncol.

[22] Singh M, Loftus T, Webb E, Benencia F (2016). Minireview: regulatory T cells and ovarian cancer. Immunol Invest.

[23] Knutson KL, Maurer MJ, Preston CC, Moysich KB, Goergen K, Hawthorne KM (2015). Regulatory T cells, inherited variation, and clinical outcome in epithelial ovarian cancer. Cancer Immunol Immunother.

[24] Hoffmann JC, Schön MP (2021). Integrin α_E_(CD103)β_7_ in epithelial cancer. Cancers.

[25] Laumont CM, Wouters MCA, Smazynski J, Gierc NS, Chavez EA, Chong LC (2021). Single-cell profiles and prognostic impact of tumor-infiltrating lymphocytes coexpressing CD39, CD103, and PD-1 in ovarian cancer. Clin Cancer Res.

[26] Webb JR, Wick DA, Nielsen JS, Tran E, Milne K, McMurtrie E (2010). Profound elevation of CD8^+^ T cells expressing the intraepithelial lymphocyte marker CD103 (alpha_E_/beta_7_ Integrin) in high-grade serous ovarian cancer. Gynecol Oncol.

[27] Bösmüller HC, Wagner P, Peper JK, Schuster H, Pham DL, Greif K (2016). Combined immunoscore of CD103 and CD3 identifies long-term survivors in high-grade serous ovarian cancer. Int J Gynecol Cancer.

[28] Dong HP, Elstrand MB, Holth A, Silins I, Berner A, Trope CG (2006). NK- and B-cell infiltration correlates with worse outcome in metastatic ovarian carcinoma. Am J Clin Pathol.

[29] Wei X, Jin Y, Tian Y, Zhang H, Wu J, Lu W (2016). Regulatory B cells contribute to the impaired antitumor immunity in ovarian cancer patients. Tumour Biol.

[30] Nielsen JS, Sahota RA, Milne K, Kost SE, Nesslinger NJ, Watson PH (2012). CD20^+^ tumor-infiltrating lymphocytes have an atypical CD27-memory phenotype and together with CD8^+^ T cells promote favorable prognosis in ovarian cancer. Clin Cancer Res.

[31] Truxova I, Kasikova L, Hensler M, Skapa P, Laco J, Pecen L (2018). Mature dendritic cells correlate with favorable immune infiltrate and improved prognosis in ovarian carcinoma patients. J Immunother Cancer.

[32] Labidi-Galy SI, Sisirak V, Meeus P, Gobert M, Treilleux I, Bajard A (2011). Quantitative and functional alterations of plasmacytoid dendritic cells contribute to immune tolerance in ovarian cancer. Cancer Res.

[33] Ning F, Cole CB, Annunziata CM (2021). Driving immune responses in the ovarian tumor microenvironment. Front Oncol.

[34] Nersesian S, Glazebrook H, Toulany J, Grantham SR, Boudreau JE (2019). Naturally killing the silent killer: NK cell-based immunotherapy for ovarian cancer. Front Immunol.

[35] Romee R, Foley B, Lenvik T, Wang Y, Zhang B, Ankarlo D (2013). NK cell CD16 surface expression and function is regulated by a disintegrin and metalloprotease-17 (ADAM17). Blood.

[36] Larionova I, Tuguzbaeva G, Ponomaryova A, Stakheyeva M, Cherdyntseva N, Pavlov V (2020). Tumor-associated macrophages in human breast, colorectal, lung, ovarian and prostate cancers. Front Oncol.

[37] Hoover AA, Hufnagel DH, Harris W, Bullock K, Glass EB, Liu E (2020). Increased canonical NF-kappaB signaling specifically in macrophages is sufficient to limit tumor progression in syngeneic murine models of ovarian cancer. BMC Cancer.

[38] Nowak M, Klink M (2020). The role of tumor-associated macrophages in the progression and chemoresistance of ovarian cancer. Cells.

[39] Osborn G, Stavraka C, Adams R, Sayasneh A, Ghosh S, Montes A (2022). Macrophages in ovarian cancer and their interactions with monoclonal antibody therapies. Clin Exp Immunol.

[40] Leary A, Tan D, Ledermann J (2021). Immune checkpoint inhibitors in ovarian cancer: where do we stand?. Ther Adv Med Oncol.

[41] Imai Y, Hasegawa K, Matsushita H, Fujieda N, Sato S, Miyagi E (2018). Expression of multiple immune checkpoint molecules on T cells in malignant ascites from epithelial ovarian carcinoma. Oncol Lett.

[42] Pawłowska A, Suszczyk D, Tarkowski R, Paduch R, Kotarski J, Wertel I (2020). Programmed death-1 receptor (PD-1) as a potential prognosis biomarker for ovarian cancer patients. Cancer Manag Res.

[43] Darb-Esfahani S, Kunze CA, Kulbe H, Sehouli J, Wienert S, Lindner J (2016). Prognostic impact of programmed cell death-1 (PD-1) and PD-ligand 1 (PD-L1) expression in cancer cells and tumor-infiltrating lymphocytes in ovarian high grade serous carcinoma. Oncotarget.

[44] Xu M, Zhang B, Zhang M, Liu Y, Yin FL, Liu X (2016). Clinical relevance of expression of B7-H1 and B7-H4 in ovarian cancer. Oncol Lett.

[45] Cai J, Wang D, Zhang G, Guo X (2019). The role Of PD-1/PD-L1 axis in Treg development and function: Implications for cancer immunotherapy. Onco Targets Ther.

