# Supplementary material for: The prognostic significance of tumor-immune microenvironment in ascites of patients with high-grade serous carcinoma
Source: Radiol Oncol. 2023 Nov 30;57(4):493–506. doi: 10.2478/raon-2023-0046 (PMC10690755; doi:10.2478/raon-2023-0046)
Supplement: Supplementary file 1 — Supplementary Material Details [file raon-2023-0046_sm.pdf]

sciendo Radiology and Oncology | Ljubljana | Slovenia | www.radioloncol.com
RADIOLOGY AND ONCOLOGY

The prognostic significance of tumor-immune microenvironment in ascites of patients with high-grade serous carcinoma

Simona Miceska, Erik Skof, Simon Bucek,Cvetka Grasic Kuhar, Gorana Gasljevic, Spela Smrkolj, Veronika Kloboves Prevodnik

doi: 10.2478/raon-2023-0046

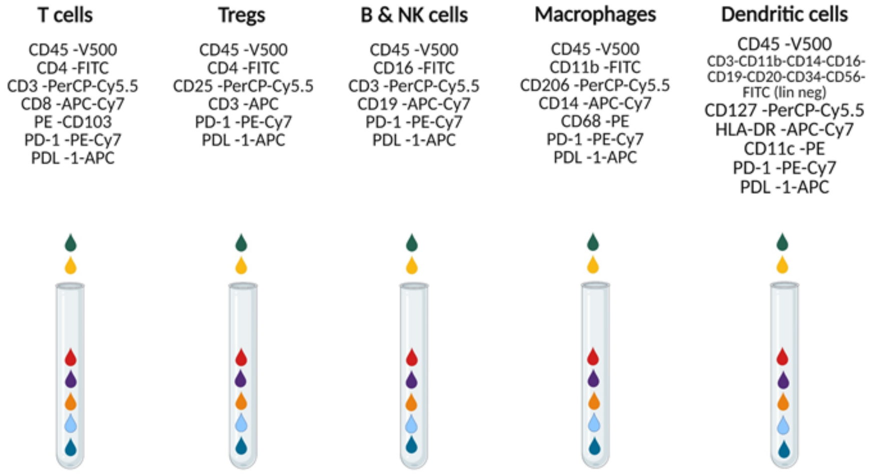

SUPPLEMENTARY FIGURE 1. Schematic description of the antibody combinations used for identifying immune cells in five separate tubes. Created with BioRender.com.

APC = allophycocyanine; FITC = fluorescein isothiocyanate; PE = phycoerythrin; PE-Cy7 = phycoerythrin-cyanine7; PerCP = peridinin chlorophyll protein; V500 = violet-500

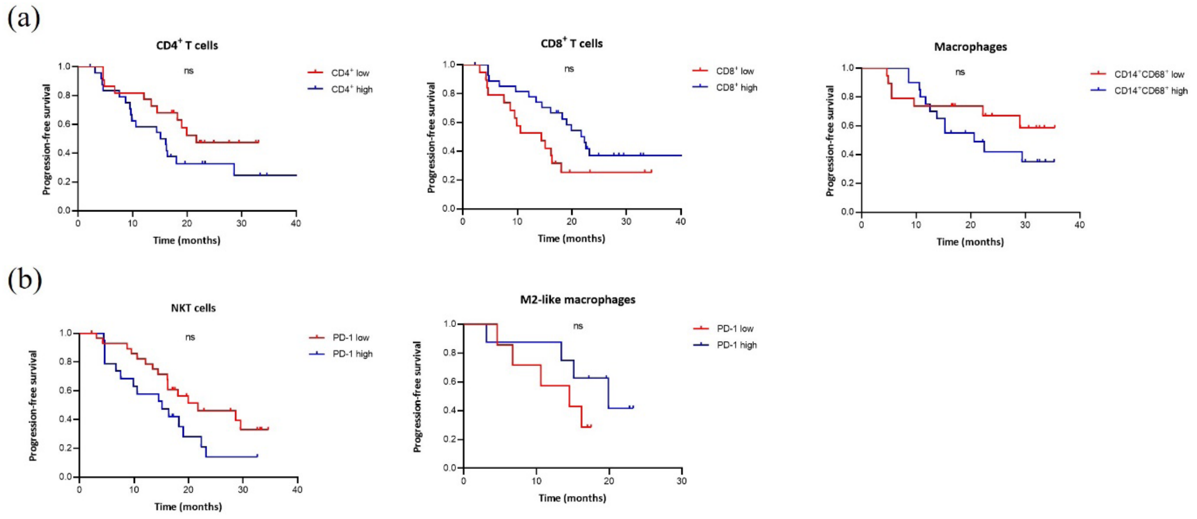

SUPPLEMENTARY FIGURE 2. Kaplan-Meier curves for progression-free survival (PFS) of high-grade serous carcinoma (HGSC) patients. (**A**) PFS curves indicating a trend for patients stratified as having low or high percentages of immune cells. (**B**) Overall survival (OS) curves indicate a trend for patients stratified as having a high or low expression of PD-1 on immune cells. Cut-off values were based on the population median.

ns = no significant

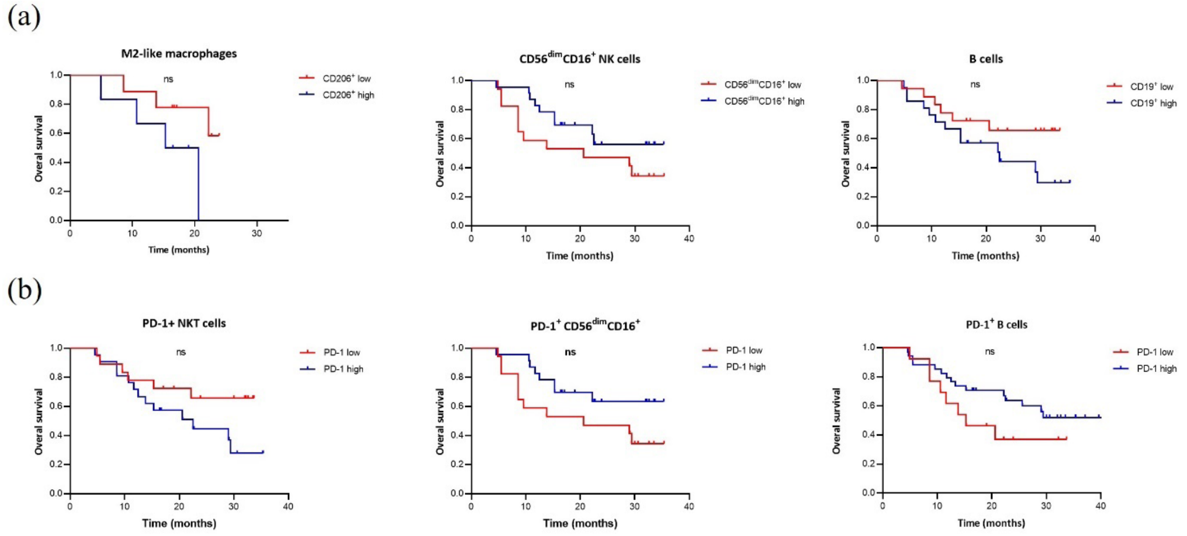

SUPPLEMENTARY FIGURE 3. Kaplan-Meier curves for overall survival (OS) of high-grade serous carcinoma (HGSC) patients. (**A**) OS curves indicating a trend for patients stratified as having low or high percentages of immune cells. (**B**) OS curves indicate a trend for patients stratified as having a high or low expression of PD-1 on immune cells. Cut-off values were based on the population median.

ns = no significant

SUPPLEMENTARY TABLE 1. Antibodies used for detection of the immune cell populations/subsets in the ascites

No.	Antigen	Conjugate	Volume per 100 µm	Clone	Reaction	Cat. No.	Manufacturer
1	CD3	APC-Cy7	3	SK7	membrane	341110	BD
2	CD3	FITC	3	SK7	membrane	345763	BD
3	CD3	PerCp-Cy5.5	3	SK7	membrane	332771	BD
4	CD4	FITC	3	SK3	membrane	345768	BD
5	CD8	APC-Cy7	2	SK1	membrane	348813	BD
6	CD11b	FITC	10	Bear-1	membrane	IM0530U	Beckman Coulter
7	CD11c	PE	5	S-HCL-3	membrane	333149	BD
8	CD14	APC-Cy7	2	MφP9	membrane	561709	BD
9	CD14	FITC	3	M5E2	membrane	301804	BioLegend
10	CD16	FITC	3	B73.1	membrane	360716	BioLegend
11	CD19	FITC	3	SJ25C1	membrane	363008	BioLegend
12	CD19	PerCP-Cy5.5	5	SJ25C1	membrane	332780	BD
13	CD20	APC-Cy7	5	L27	membrane	335829	BD
14	CD20	FITC	10	L27	membrane	340673	BD
15	CD25	PerCP-Cy5.5	5	M-A251	membrane	356112	BioLegend
16	CD34	FITC	3	8G12	membrane	345801	BD
17	CD45	V500	2	HI30	membrane	560777	BD
18	CD56	PE	10	NCAM16.2	membrane	345812	BD
19	CD56	FITC	5	NCAM16.2	membrane	345811	BD
20	CD68	PE	5	Y1/82A	cytoplasmic	333808	BioLegend
21	CD103	PE	5	Ber-ACT8	membrane	350206	BioLegend
22	CD123	PerCP-Cy5.5	5	7G3	membrane	558714	BD Pharmingen
23	CD127	PE	5	hIL-7R-M21	membrane	557938	BD Pharmingen
24	CD206	PerCP-Cy5.5	5	C068C2	membrane	141716	BioLegend
25	HLA-DR	APC-Cy7	5	L243	membrane	307618	BioLegend
26	PD-1	PE-Cy7	5	EH12.1	membrane	561272	BD Pharmingen
27	PD-L1	APC	5	MIH1	membrane	563741	BD Pharmingen

SUPLEMENTARY TABLE 2. Detailed description of the patients stratified with high and low percentages of immune cells and PD-1 expression in HGSC ascites

ICs	Total N of patients analyzed for ICs	Patients with low percentages of ICs	Patients with high percentages of immune cells	Patients analyzed for PD-1 expression on ICs	Patients with low PD-1 expression	Patients with high PD-1 expression
	N	N (%)	N (%)	N	N (%)	N (%)
$CD3^+$	47	22 (47)	25 (53)	39	18 (46)	21 (54)
$CD4^+$	47	22 (47)	25 (53)	39	19 (49)	20 (51)
$CD8^+$	47	19 (40)	28 (60)	39	18 (46)	21 (54)
Tregs	39	16 (41)	23 (59)	39	20 (51)	19 (49)
$CD103^+CD3^+$	47	19 (40)	28 (60)	39	18 (46)	21 (54)
$CD103^+CD4^+$	39	16 (41)	23 (59)	39	20 (51)	19 (49)
$CD103^+CD8^+$	39	16 (41)	23 (59)	39	19 (49)	20 (51)
CD8/CD4 index	47	24 (51)	23 (49)	39	19 (49)	20 (51)
NKT cells	47	19 (40)	28 (60)	39	18 (46%)	21 (54)
$CD56^{bright}CD16^-$ NK cells	39	20 (51)	19 (49)	39	25 (64%)	14 (36)
$CD56^{dim}CD16^+$ NK cells	39	16 (41)	23 (59)	39	16 (41%)	23 (59)
Macrophages	39	19 (49)	20 (51)	39	20 (51%)	19 (49)
M1-like macrophages	15	6 (40)	9 (60)	15	6 (40)	9 (60)
M2-like macrophages	15	9 (60)	6 (40)	15	8 (53%)	7 (47)
B cells	39	18 (46)	21 (54)	39	9 (23%)	30 (77)
DCs	39	9 (23)	30 (77)	39	30 (77%)	9 (23)

DCs = dendritic cells; ICs = immune cells; N = number of patients; NK = natural killer; NKT = NK T; PD-1 = programmed cell death protein 1; Tregs = regulatory T cells; % = percentages
